# A Modified Kalman Filter for Integrating the Different Rate Data of Gyros and Accelerometers Retrieved from Android Smartphones in the GNSS/IMU Coupled Navigation

**DOI:** 10.3390/s20185208

**Published:** 2020-09-12

**Authors:** Wenlin Yan, Qiuzhao Zhang, Lijuan Wang, Ying Mao, Aisheng Wang, Changsheng Zhao

**Affiliations:** 1 School of Geography, Geomatics and Planning, Jiangsu Normal University, 101 Rd. Shanghai, Xuzhou 221116, China; yanwenlin@jsnu.edu.cn (W.Y.); 3020171315@jsnu.edu.cn (Y.M.); 6020010013@jsnu.edu.cn (A.W.); zhaocs1957@126.com (C.Z.); 2 School of Environment Science and Spatial Informatics, China University of Mining and Technology, No 1, Daxue Road, Xuzhou 221116, China; qiuzhao.zhang@cumt.edu.cn

**Keywords:** GNSS/IMU coupled navigation, Kalman filter, android smartphones, IMU

## Abstract

Recent study indicates that by using the inertial measurement unit (IMU) sensors inside smartphones, we can obtain similar navigation solutions to the professional ones. However, the sampling rates of the gyros and accelerometers inside some types of smartphones are not set in the same frequencies, i.e., the gyros of “Huawei p40” are in 50 Hz while the accelerometer is 100 Hz. The conventional method is resampling the higher frequency to the lower frequency ones, which means the resampled accelerometer will lose half frequency observations. In this work, a modified Kalman filter was proposed to integrate all these different rate IMU data in the GNSS/IMU-smartphone coupled navigation. To validate the proposed method, a terrestrial test with two different types of android smartphones was done. With the proposed method, a slight improvement of the attitude solutions can be seen in the experiments under the GNSS open-sky condition, and the obvious improvement of the attitude solutions can be witnessed at the simulated GNSS denied situation. The improvements by 45% and 23% of the horizontal position accuracy can be obtained from the experiments under the GNSS outage of 50 s in a straight line and 30 s in a turning line, respectively.

## 1. Introduction

The quick development of modern miniaturization technology makes it possible to embed more high-performance sensors into smartphones, and the population of the smartphones in the mass market also gives great promotions to feedback this technology. We are focused on using the chips inside smartphones, including IMU (inertial measurement unit), GNSS, camera, etc., to develop potential professional-level works, such as high precision navigation, photogrammetry and remote sensing.

Recently, the performance of the IMU sensors inside the latest smartphones has been improved significantly, and many potential applications based on these sensors can be validated. Castro-Palacio et al. (2014) used a smartphone acceleration sensor to study the accelerated circular motions and obtained high agreement results compared with other reference methods [1]. Mourcou et al. (2015) analyzed the reliability of the clinical motion detection using the IMUs of smartphones [2]. Masiero and Vettore (2016) improved the image feature matching capacity with the simultaneously recorded image and IMU data from a smartphone [3]. Hakim et al. (2017) designed a system for detecting the fall accident for disabled people using the IMUs inside smartphones [4]. Andriamandroso et al. (2017) tried to use the smartphone’s internal IMU sensors to characterize cattle eating and ruminating behaviors, with a high detection accuracy of more than 90% [5]. Poulose et al. (2018) studied the indoor position algorithm using a Samsung Galaxy Note 8 smartphone and obtained a 0.94–2.6 m accuracy trajectory result in a floor of one building. Sun et al. (2019) used the accelerometers and gyros inside the smartphone to detect the vehicle driving event, achieving a high correct accuracy of 97.5% [6]. 

IMU modules inside the smartphones also have great potentials to improve the navigation performance coupled with the GNSS observables. Mostafa et al. (2019) used the integration of GNSS, an INS-smartphone (inertial navigation system) and other visual sensors to enhance the USV (unmanned surface vehicle) navigation system, with around 80% reduction in errors [7]. Our previous studies on the assessment of the navigation performance of the IMUs inside smartphones provided an initial discussion about the integration method of the gyros and accelerometers of smartphones [8]. 

Recently, we found the sampling rates of the gyros and accelerometers in some types of smartphones, however, are not set in the same frequencies, for instance, the gyro data of “Huawei P40” is in 50 Hz whilst the accelerometer is 100 Hz. The conventional Kalman filter used in previous work was resampling the high frequency to the lower frequency ones, i.e., the 100 Hz accelerometers data were shrunk into 50 Hz to match the lower rate gyro data, which caused the great loss of the high frequency data. In this work, the structure of the prediction process of the Kalman filter was modified to prevent the discarding of the high frequency IMU data, to seek an improvement in the GNSS/IMU-smartphone coupled navigation. In the following section the modified Kalman filter will be described in detail, after that a terrestrial test strapped with two types of smartphones is validated to test the reliabilities of the proposed method. 

## 2. The Kalman Filter for the GNSS/IMU Coupled Navigation

GNSS/IMU coupled navigation is one of the most classical navigation strategies, which can take full advantage of the GNSS and IMU system. The Kalman filter plays an essential role in the GNSS/IMU coupled algorithm. In general, the Kalman filter comprises two parts, the prediction process and the update process.

### 2.1. The Prediction Process of the Kalman Filter

In the prediction process, the gyros and the accelerometers observations are compensated, integrated and converted to calculate the navigation parameters. Taking a 15-state Kalman filter as an example, the navigation parameters vector can be written as:

(1) $$x={\left[\begin{array}{ccccc} r^{n} & v^{n} & \psi & b_{a} & b_{\omega } \end{array}\right]}^{T}{}_{15\times 1}$$

where $r^{n}$ means the position vector in the navigation frame, $v^{n}$ means the velocity vector in the navigation frame, ψ means the vector of the Euler angles that rotate from the navigation frame to the body frame, $b_{a}$ and $b_{\omega }$ mean the bias error vector of the accelerometers and gyros.

The prediction equation of the state parameters can be written with a dynamic form as:

(2) $$\dot{x}=f\left(x\right)+Gw$$

where f is the system dynamic function, w is the system noise matrix and G is a time-varying matrix.

The detail form of the position and velocity state dynamic equations can be written as [9]:

(3) $$\{\begin{array}{l} \dot{L}=\frac{v_{E}}{R_{0}+h}\\ \dot{l}=\frac{v_{E}\sec L}{R_{0}+h}\\ \dot{h}=-v_{D} \end{array}$$

and

(4) $$\{\begin{array}{l} {\dot{v}}_{N}=f_{N}-2\Omega v_{E}\sin L+\frac{v_{N}v_{D}-v_{E}^{2}\tan L}{R_{0}+h}+\xi g\\ {\dot{v}}_{E}=f_{E}+2\Omega \left(v_{N}\sin L+v_{D}\cos L\right)+\frac{v_{E}}{R_{0}+h}\left(v_{D}+v_{N}\tan L\right)-\eta g\\ {\dot{v}}_{D}=f_{D}-2\Omega v_{E}\cos L-\frac{v_{E}^{2}+v_{N}^{2}}{R_{0}+h}+g \end{array}$$

where *L*, *l* and *h* mean the latitude (in radians), longitude (in radians) and height (in meters) of the position parameters, respectively, *Ω* is the turn rate of the earth (7.2921151467e-5 rad/s), *R*_0_ is the mean radius of the Earth (6,371,008.7714 m), *ξ* and *η* represent angular deflections (in radians) in the direction of local gravity vector with respect to the local vertical owing to gravity anomalies (g), (*f_N_*, *f_E_*, *f_D_*) mean the converted accelerometer observations in the navigation frame along the North, East and Down direction, respectively, which can be calculated as:

(5) $$\left[\begin{array}{c} f_{N}\\ f_{E}\\ f_{D} \end{array}\right]=C_{b}^{n}\left[\begin{array}{c} f_{x}^{b}\\ f_{y}^{b}\\ f_{z}^{b} \end{array}\right]$$

where ($f_{x}^{b}$, $f_{y}^{b}$, $f_{z}^{b}$) mean the accelerometer observations (in m/s^2^), and $C_{b}^{n}$ is the direct cosine matrix for the transformation from the body frame to the navigation frame, calculated with the Euler angles as [8]:

(6) $$C_{b}^{n}=\left[\begin{array}{ccc} \cos\theta \cos\psi & -\cos\phi \sin\psi +\sin\phi \sin\theta \cos\psi & \;\sin\phi \sin\psi +\cos\phi \sin\theta \cos\psi \\ \cos\theta \sin\psi & \;\cos\phi \cos\psi +\sin\phi \sin\theta \sin\psi & -\sin\phi \cos\psi +\cos\phi \sin\theta \sin\psi \\ -\sin\theta & \sin\phi \cos\theta & \cos\phi \cos\theta \end{array}\right]$$

The detail form of the Euler angle state dynamic equations can be written as [9]:

(7) $$\{\begin{array}{l} \dot{\phi }=\left(\omega_{y}\sin\phi +\omega_{z}\cos\phi \right)\tan\theta +\omega_{x}\\ \dot{\theta }=\omega_{y}\cos\phi -\omega_{z}\sin\phi \\ \dot{\psi }=\left(\omega_{y}\sin\phi +\omega_{z}\cos\phi \right)\sec\theta \end{array}$$

The direct cosine matrix $C_{b}^{n}$ can also be calculated by the quaternions ***q*** = [*a b c d*]^T^ as:

(8) $$C_{b}^{n}=\left[\begin{array}{ccc} a^{2}+b^{2}-c^{2}-d^{2} & 2\left(bc-ad\right) & 2\left(bd+ac\right)\\ 2\left(bc+ad\right) & \;a^{2}-b^{2}+c^{2}-d^{2} & 2\left(cd-ab\right)\\ 2\left(bd-ac\right) & 2\left(cd+ab\right) & a^{2}-b^{2}-c^{2}+d^{2} \end{array}\right]$$

and the quaternions dynamic equations is:

(9) $$\{\begin{array}{l} \dot{a}=-0.5\left(b\omega_{x}+c\omega_{y}+d\omega_{z}\right)\\ \dot{b}=0.5\left(a\omega_{x}-d\omega_{y}+c\omega_{z}\right)\\ \dot{c}=0.5\left(d\omega_{x}+a\omega_{y}-b\omega_{z}\right)\\ \dot{d}=-0.5\left(c\omega_{x}-b\omega_{y}-a\omega_{z}\right) \end{array}$$

where (*ω*_x_, *ω*_y_, *ω*_z_) (in rad/s) represent the turn rate of the body frame with respect to the navigation frame in body axes, which can be notated as a vector as $\omega_{nb}^{n}$ that is calculated by:

(10) $$\omega_{nb}^{n}=\omega_{ib}^{b}-C_{n}^{b}\left(\omega_{ie}^{n}+\omega_{en}^{n}\right)$$

where$\;\omega_{ib}^{b}$ is the observations of the gyros (in rad/s), $C_{n}^{b}$ is the transpose of $C_{b}^{n}$, $\omega_{ie}^{n}$ is the turn rate of the Earth in the local geographic frame, which can be calculated as:

(11) $$\omega_{ie}^{n}={\left[\begin{array}{ccc} \Omega \cos L & 0 & -\Omega \end{array}\sin L\right]}^{T}$$

and $\omega_{en}^{n}$ in Equation (10) is the turn rate of the local geographic frame with respect to the Earth-fixed frame, which can be calculated by:

(12) $$\omega_{en}^{n}={\left[\begin{array}{ccc} \frac{v_{E}}{R_{0}+h} & \frac{-v_{N}}{R_{0}+h} & \frac{-v_{E}\tan L}{R_{0}+h} \end{array}\right]}^{T}$$

The biases of the gyros and accelerometers are assumed to be constant, then the dynamic equations of these sensors’ biases can be written as:

(13) $$\{\begin{array}{c} {\dot{b}}_{a}=0\\ {\dot{b}}_{\omega }=0 \end{array}$$

In a non-linear expression, Equation (2) can be deduced as:

(14) $$x_{i|i-1}=x_{i-1}+\int_{t_{i-1}}^{t_{i}}f\left(x\right)dt$$

Substituting f(x) in Equation (14) with Equations (3)–(13), the state parameters can be predicted by the integral of the IMU’s observations with time.

After the prediction of the state parameters in Equation (1), the corresponding errors need to be estimated. The vector of the state errors can be written as:

(15) $$\delta x={\left[\begin{array}{ccccc} \delta r^{n} & \delta v^{n} & \delta \phi & \delta b_{a} & \delta b_{\omega } \end{array}\right]}^{T}{}_{15\times 1}$$

The prediction process of the state errors and the corresponding covariance matrix can be deduced as [10]:

(16) $$\{\begin{array}{l} \delta x_{i|i-1}=\phi_{i|i-1}\delta x_{i-1}\\ P_{i|i-1}=\phi_{i|i-1}P_{i-1}\phi_{i|i-1}^{T}+Q_{i} \end{array}$$

where $\phi_{i|i-1}$ is the prediction of the system matrix, which can be calculated by:

(17) $$\phi_{i|i-1}\approx I_{15\times 15}+F_{i-1}\Delta t+\frac{1}{2!}{\left(F_{i-1}\Delta t\right)}^{2}+\frac{1}{3!}{\left(F_{i-1}\Delta t\right)}^{3}$$

where F is the system dynamic matrix, and the detail form can be found in the documents of [9,11,12]. 

$Q_{i}$ in Equation (16) is the process noise matrix, which can be formed as [10,12]:

(18) $$Q_{i}\approx G_{i}Q\Delta tG_{i}^{T}$$

where ***G*** is the design matrix for the noise of the IMU observations, which can be deduced as:

(19) $$G={\left[\begin{array}{cc} 0 & 0\\ C_{b}^{n} & 0\\ 0 & -C_{b}^{n}\\ 0 & 0\\ 0 & 0 \end{array}\right]}_{15\times 6}$$

and ***Q*** in Equation (18) is the Spectral Density Matrix of the gyros and accelerometers observations [10,11]:

(20) $$Q_{6\times 6}=diag\left(\begin{array}{cccccc} \sigma_{ax}^{2} & \sigma_{ay}^{2} & \sigma_{az}^{2} & \sigma_{\omega x}^{2} & \sigma_{\omega y}^{2} & \sigma_{\omega z}^{2} \end{array}\right)$$

where *σ_a_* and *σ*_ω_ are the standard deviation of the accelerometers and gyros, respectively, which can be characterized by the noise of the IMUs that could be found in the manufacture document.

In general, the prediction processes of the states of the position, velocity, attitude and the sensor bias are accomplished via Equation (14), which is filled by Equations (3), (4), (7) and (13), respectively, while the corresponding errors and their covariance prediction processes are Equation (16).

### 2.2. The Update Process of the Kalman Filter

During the running of the prediction process, the update process can be trigged in the case of the GNSS observations happening. For the loosely coupled strategy, the update equations can be taken as:

(21) $$\{\begin{array}{l} K_{i}=P_{i/i-1}H_{i}{}^{T}{\left[H_{i}P_{i/i-1}H_{i}{}^{T}+R_{i}\right]}^{-1}\\ \delta x_{i}=\delta x_{i-1}+K_{i}\left[\delta z_{i}-H_{i}\delta x_{i|i-1}\right]\\ P_{i}=\left[I-K_{i}H_{i}\right]P_{i/i-1} \end{array}$$

where ***K***_15×15_ is the Kalman gain matrix, ***R***_3×3_ is the GNSS covariance matrix, *δ**z*** is the update observation vector of the state errors—in our work referring to the delta position calculated through the differences between GNSS observations and system predictions, which can be written as:

(22) $$\delta z_{i}=z_{i}-H_{i}x_{i|i-1}$$

where ***z****_i_* is the GNSS observations: $z_{i}={\left[\begin{array}{ccc} L & l & h \end{array}\right]}_{gnss,i,\;3\times 1}^{T}$, and

(23) $$H_{i}={\left[\begin{array}{ccccc} 1 & 0 & 0 & \cdots & \cdots \\ 0 & 1 & 0 & \cdots & \cdots \\ 0 & 0 & 1 & \cdots & \cdots \end{array}\right]}_{3\times 15}$$

The GNSS observation vector ***z****_i_* can be expended as ${\left[\begin{array}{cccccc} L & l & h & v_{N} & v_{E} & v_{D} \end{array}\right]}_{i,6\times 1}^{T}$, if there are reliable velocity solutions retrieved from Doppler observations, and the line number of ***H*** matrix becomes 6.

### 2.3. Advanced Developments of the Kalman Filter in the GNSS/IMU Coupled Navigation

In order to get the more accurate and robust solutions from the GNSS/IMU coupled navigation, the form of the conventional Kalman filter has been developed from different aspects.

Some developed strategies modified the update process, i.e., the tightly and ultra-tightly Kalman filter introduces the GNSS raw pseudorange and carrier observations as the update information; the Kalman filter that introduces odometer, vision-camera, or other exterior arguments for the GNSS/INS coupled navigation; the non-holonomic constrain method uses the zero velocity in the side and vertical direction of the vehicle, as the update information of the Kalman filter to limit the growth of the system errors. These methods optimize the structure of the Kalman filter to involve more update information to the navigation system, which makes the system keep running even under the GNSS challenge environment.

Other strategies, such as the adaptive and robust Kalman filter, are focused on the balance of the contributions of the prediction process and the update process, by way of introducing the scale factor *α* and *β* into the spectral density matrix ***Q*** in Equation (20) and the GNSS covariance matrix ***R*** in Equation (21), respectively.

Few development strategies make changes to the prediction process, since the observations of the gyros and accelerometers from the IMU are the main input of the system dynamic equation, as can be seen in Equations (5) and (10), and the sampling rate of the gyros and accelerometers normally is set as the same frequency.

## 3. The Modified Kalman Filter for Integrating the Different Rate Data of Gyros and Accelerometers

As stated in the previous section, the data of the gyros and accelerometers can be integrated to calculate the position, velocity and attitude parameters in the predict process, and the data rate of the two sensors needs to be identical in the conventional Kalman filter.

**Figure 1:**
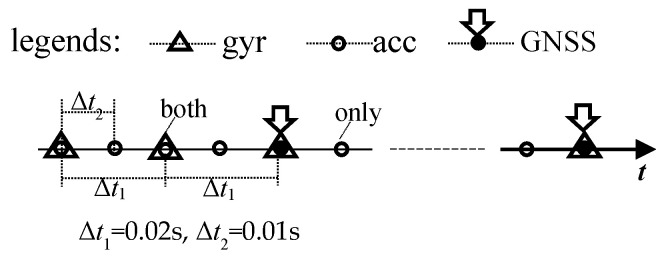
The sequence of the inertial measurement unit (IMU) data of the “Huawei P40”.

However, we found the data rates of the two sensors are different in some types of smartphones, i.e., the data frequency of gyros of the “Huawei P40” is 50 Hz while that of accelerometers is 100 Hz. As shown in Figure 1, where the triangles mean the gyros observations with the time interval as Δ*t*_1_ = 0.02 s, and the hollow circles mean the accelerometer observations with the double frequency samplings compared to the gyros, the time interval for this is Δ*t*_2_ = 0.01 s.

Theoretically, the data of the accelerometers can be resampled into the same frequency as the gyros to apply the conventional Kalman filter. This resampling method is not the optimal way to integrate the data since half of the accelerometers are abandoned. In this work, we make the changes of structure of the prediction process of the Kalman filter to include all the data of the gyros and accelerometers.

From Figure 1, we can see that there are two different epochs of the data sequence, one is with the observations of both gyros and accelerometers tagged with the symbol of a circle inside a triangle, and we named it as “both” epoch; the other one is only with the accelerometers, with the symbol of a simple circle, and named as “only” epoch.

At first, the conventional Kalman filter can be applied at the “both” epoch, but the item of “Δ*t*” in Equation (14) should be taken with different values for the gyros and accelerometers—take the “Huawei P40” as an example, “Δ*t*_1_” 0.02 s is for the conventional Kalman filter and the accelerometers “Δ*t*_2_” 0.01 s is for the proposed method.

Secondly, at the “only” epoch the attitude predication using Equation (7) is not taken since there are no gyro observations. Subsequently, a new noise matrix base using Equation (18) was designed as:

(24) $$Q_{i}\approx G_{i}^{\prime }Q^{\prime }\Delta t_{2}{G^{\prime }}_{i}^{T}$$

where

(25) $$G^{\prime }={\left[\begin{array}{c} 0\\ C_{b}^{n}\\ 0\\ 0\\ 0 \end{array}\right]}_{15\times 3}$$

and

(26) $${Q^{\prime }}_{3\times 3}=diag\left(\begin{array}{ccc} \sigma_{ax}^{2} & \sigma_{ay}^{2} & \sigma_{az}^{2} \end{array}\right)$$

Then, the modified Kalman filter with the two steps above, can be adopted to the data sequence shown in Figure 1.

**Figure 2:**
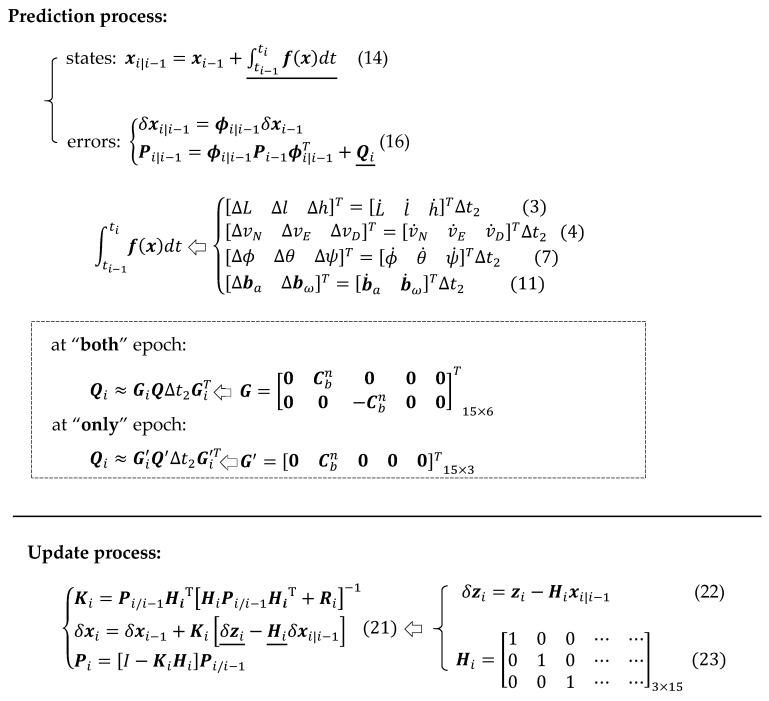
Summary of the process procedures of the GNSS/IMU-P40 coupled navigation.

In a short summary, to include the high rate data of the accelerometers, the prediction process was performed twice more than the conventional Kalman filter, and the covariance matrix ***Q*** was modified according to the IMU sampling “epoch” status; the process procedures can be summarized as Figure 2. This proposed method mainly works on the prediction process of the Kalman filter to include more IMU data, which differs from the other modification strategies that were stated in the previous section, such as GNSS/IMU tightly coupled, GNSS/IMU/Vision or non-holonomic constrains.

## 4. Test and Results

**Figure 3:**
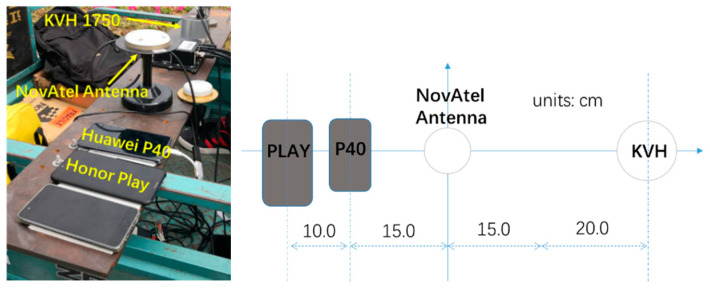
The physical setup of the devices used in the terrestrial test.

In order to test the reliability of the proposed method, a terrestrial test with two smartphones was carried out on the morning of 27 June 2020. These two mobile phones, “Huawei P40” and “Honor Play” made by Huawei Technologies co. LTD. (Shenzhen, China), were strapped down on the top of an iron board, which was fixed onto a motor tricycle as can be seen in Figure 3. Beside the smartphones, a high-grade fiber IMU, “KVH 1750” (200 Hz) made by KVH Industries, Inc. (Middletown 06457, USA), was taken as the comparing reference. Besides the IMU, a dual-frequency GNSS receiver (1 Hz), “NovAtel OEM6” made by NovAtel Inc. (Calgary, Canada), that was put on the same platform, as well as a GNSS reference nearby, was also involved to provide differential GNSS solutions.

### 4.1. The Reference Solutions of the GNSS/KVH Coupled Navigations

**Figure 4:**
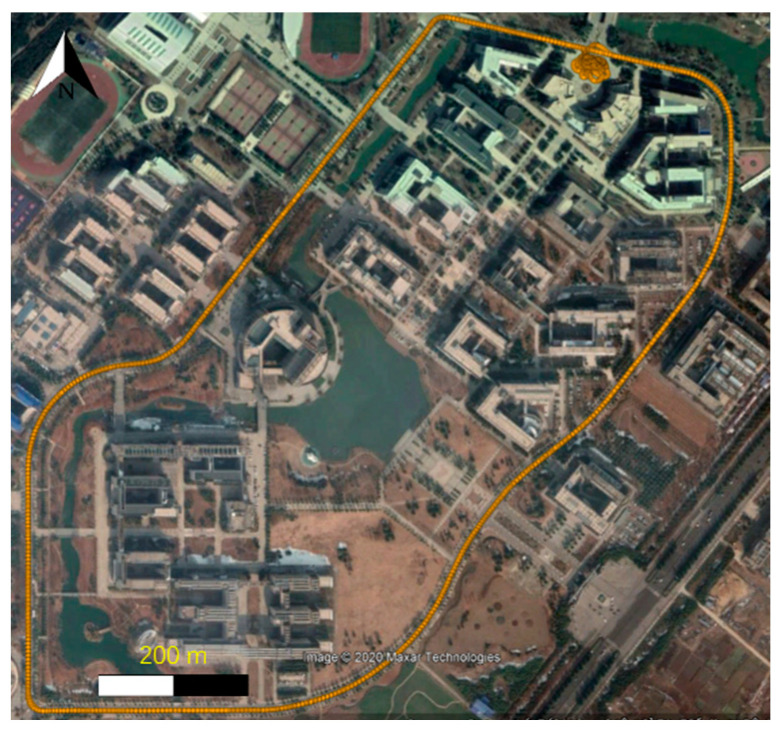
The trajectory projected in Google Earth.

This test was carried out at the campus of China University of Mining and Technology, the trajectory projected in Google Earth is as shown in Figure 4. The average velocity of the motor tricycle was around 4.5 m/s, and the time duration of this test is around 50 min.

The GNSS, GNSS/KVH coupled navigation solutions were estimated by the NovAtel “Inertial Explorer” (IE) software in a LC (loosely coupled) mode, and the profile was set as the manufacture “NovAtel SPAN Ground (KVH 1750)”. The position, velocity and attitude information from the GNSS/KVGH were taken as the reference to compare with those calculated from the GNSS/IMU-smartphone coupled navigation.

In this work, only the GNSS data from the NovAtel receiver outside the smartphones were taken to provide the GNSS update information, rather than directly using that which were logged from the GNSS chips inside the smartphones. It is due to this, that the precise positioning technology using the smartphone internal GNSS modules is still a challenging task, and in order to get rid of the affection of the different oriented GNSS information, only the NovAtel GNSS solutions were used.

### 4.2. The GNSS/IMU-Smartphone Coupled Navigation

Firstly, to record the raw IMU observation from the two smartphones, we developed the app to log these data based on the Google SDK and API via the Android Studio. The sampling data of the raw data of the android smartphones can be configured with four options:

- “SENSOR_DELAY_FASTEST” -> obtain sensor data as fast as possible;
- “SENSOR_DELAY_GAME”-> rate suitable for games;
- “SENSOR_DELAY_UI”-> rate suitable for the user interface;
- “SENSOR_DELAY_NORMAL”-> rate suitable for screen orientation changes.

In our test, the data collecting app was set with the model of “SENSOR_DELAY_GAME”. However, the IMU data were logged at different rates, for instant, the data gyro data rate of “P40” is in 50 Hz but the accelerometer is in 100 Hz, and both of gyros and accelerometers are in 100 Hz in “Honor Play”. Therefore, in order to include all the accelerometers data of “P40” and test the reliability of the proposed modified Kalman filter, we designed seven experiments of the data process of the GNSS/IMU-smartphone coupled navigation, which are listed as:

1. “Honor Play” 100 Hz gyros and 100 Hz accelerometers in GNSS open-sky condition.
2. “Huawei P40” 50 Hz gyros and 50 Hz accelerometers in GNSS open-sky condition.
3. “Huawei P40” 50 Hz gyros and 100 Hz accelerometers in GNSS open-sky condition.
4. “Huawei P40” 50 Hz gyros and 50 Hz accelerometers in a 50 s GNSS denying condition (straight line).
5. “Huawei P40” 50 Hz gyros and 100 Hz accelerometers in a 50 s GNSS denying condition (straight line).
6. “Huawei P40” 50 Hz gyros and 50 Hz accelerometers in a 30 s GNSS denying condition (turning).
7. “Huawei P40” 50 Hz gyros and 100 Hz accelerometers in a 30 s GNSS denying condition (turning).

#### 4.2.1. The Navigation Performance under the Open-Sky Conditions

For the first and second experiments, in the GNSS open-sky condition and with the gyros and accelerometers in the same sampling rate, the conventional Kalman filter can be directly applied according to Figure 2. During the prediction process of the Kalman filter, the correct specification of the IMU bias and white noise level in Equations (16) and (21) are critical in order to obtain reasonable solutions. The manufacture specification of the reference IMU “KVH 1750” and the IMUs in the two smartphones are listed in Table 1.

Beside the conventional Kalman filter, the proposed Kalman filter was also applied in the third experiment to include the high rate data of the accelerometers in “Huawei P40”.

**Figure 5:**
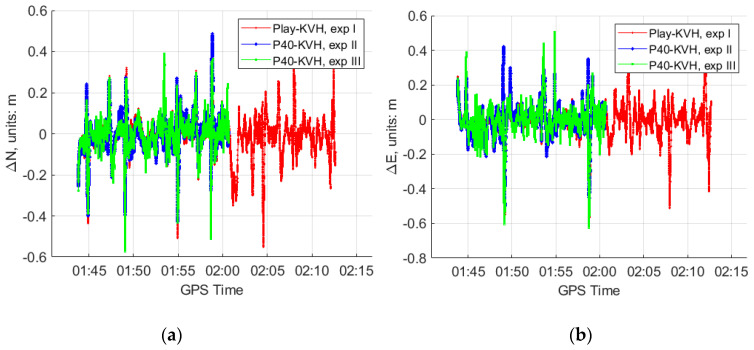

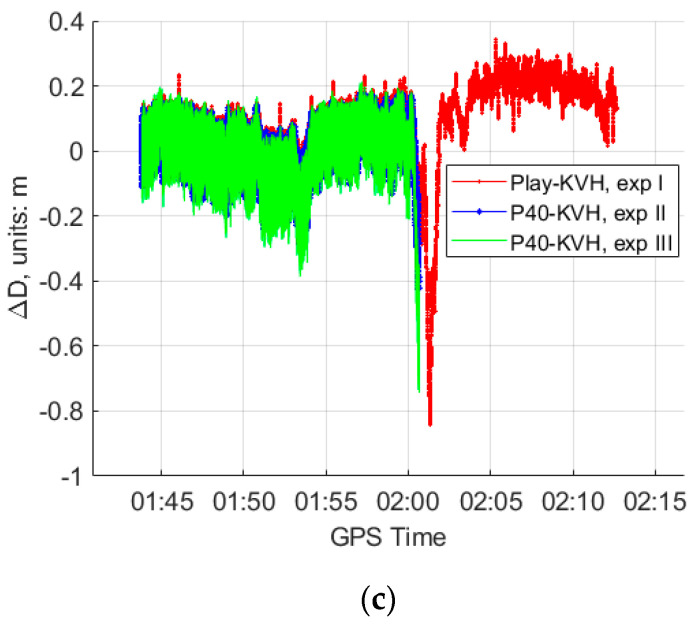
The position differences of the GNSS/IMU coupled solutions between the two smartphones (our program in a 15-state Kalman filter and the “KVH 1750” (IE software) in GNSS open-sky condition at experiment I, II and III. (**a**) Position differences in the North direction; (**b**) position differences in the East direction; (**c**) position differences in the Down direction.

**Figure 6:**
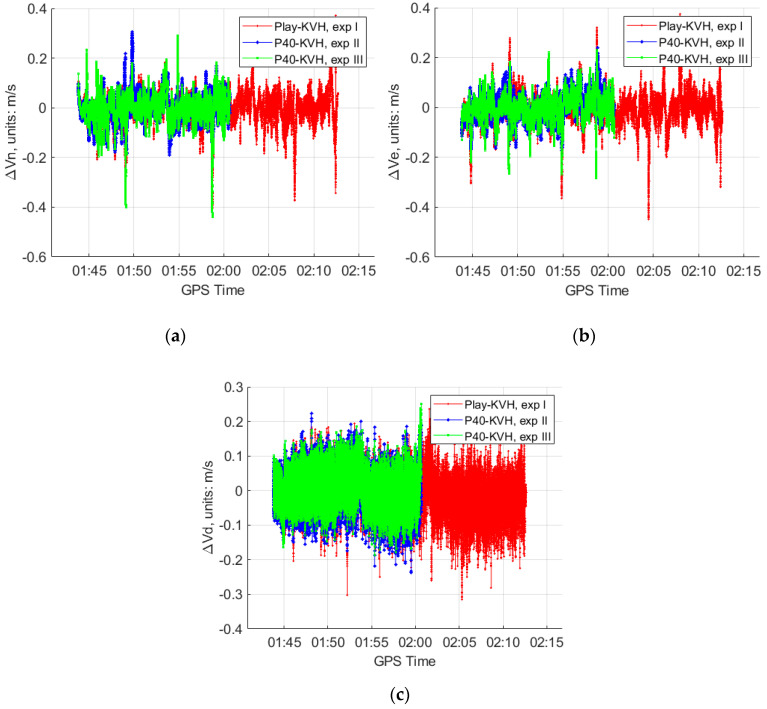
The velocity differences of the GNSS/IMU coupled solutions between the two smartphones (our program in a 15-state Kalman filter) and the “KVH 1750” (IE software) in GNSS open-sky condition at experiment I, II and III. (**a**) Velocity differences in the North direction; (**b**) velocity differences in the East direction; (**c**) velocity differences in the Down direction.

**Figure 7:**
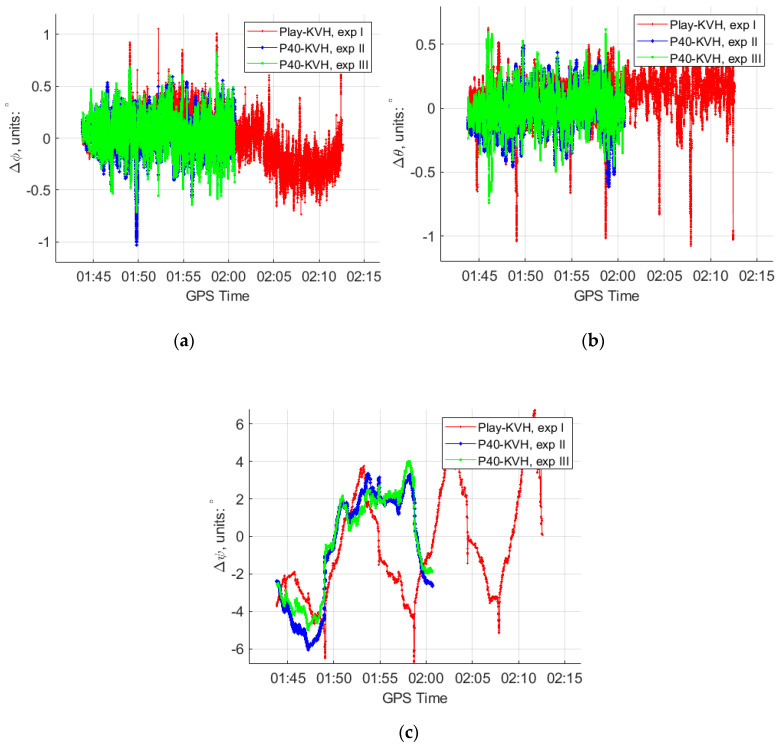
The attitude differences of the GNSS/IMU coupled solutions between the two smartphones (our program in a 15-state Kalman filter) and the “KVH 1750” (IE software) in GNSS open-sky condition at experiment I, II and III. (**a**) Roll differences; (**b**) pitch differences; (**c**) yaw differences.

Then, the position, velocity and attitude parameters can be estimated by the GNSS/IMU-smartphones for the three experiments. The differences of the solutions between the smartphones and the reference “KVH 1750” are shown in Figure 5, Figure 6 and Figure 7.

The RMS (root mean square) statistics of the comparison differences between the position, velocity and attitude solutions of the three experiments are shown in Table 2.

From Figure 5, Figure 6 and Figure 7 and Table 2, we can see that, in the GNSS open-sky condition:

1. The GNSS/IMU-smartphones coupled navigation can obtain reasonable solutions, for instance, the position differences with respect to the reference are around 0.1 m, the velocity differences are around 0.05 m/s, the roll and pith differences are around 0.2°, and the yaw differences are around 3°.
2. After introducing the high rate data of the accelerometers to the modified Kalman filter, the GNSS/IMU-P40 coupled navigation does not show the obvious improvements at the position and velocity solutions. However, slight improvements can be witnessed at the attitude solutions in the experiment III, as shown in Table 2.

#### 4.2.2. The Navigation Performance in the Simulated GNSS-Denied Environment

**Figure 8:**
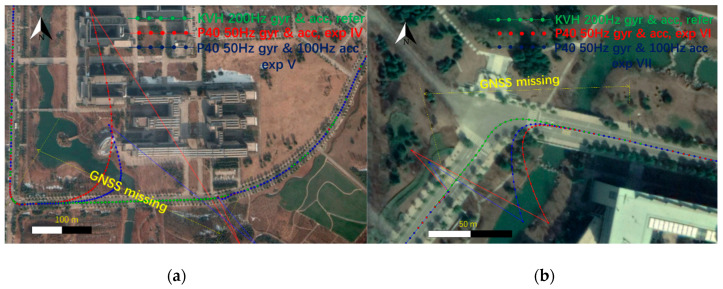
The trajectory of experiments IV to VII projected in Google Earth. The green reference trajectory is from the estimation of GNSS/KVH by the NovAtel IE software, under the GNSS open-sky conditions; the red trajectory is from the estimation of GNSS/IMU-P40 with the 50 Hz gyros and accelerometers by the conventional 15-state Kalman filter, under the GNSS-denied environment; the blue trajectory is from the estimation of GNSS/IMU-P40 with the 50 Hz gyros and 100 Hz accelerometer by our modified Kalman filter, under the GNSS-denied environment. (**a**) The 50 s GNSS observables missing in straight line; (**b**) the 30 s GNSS observables missing in turn trajectory line.

In order to test the reliability of the modified Kalman filter in a more challenging environment, 50 s GNSS observables in straight trajectory line and 30 s GNSS observables in a turn trajectory line were removed to simulate the GNSS-denied situation as shown in Figure 8.

**Figure 9:**
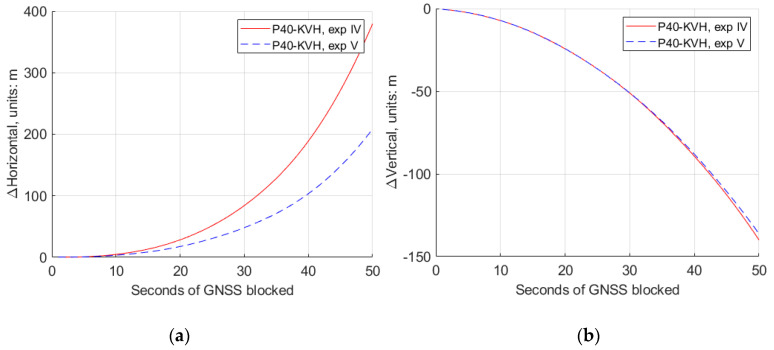
The horizontal and vertical position differences between GNSS/IMU-P40 (our program in a 15-state Kalman filter) and GNSS/KVH coupled navigation (IE software), with the conventional Kalman filter (experiment IV) and the modified Kalman filter (experiment V). (**a**) Horizontal position differences; (**b**) vertical position differences.

**Figure 10:**
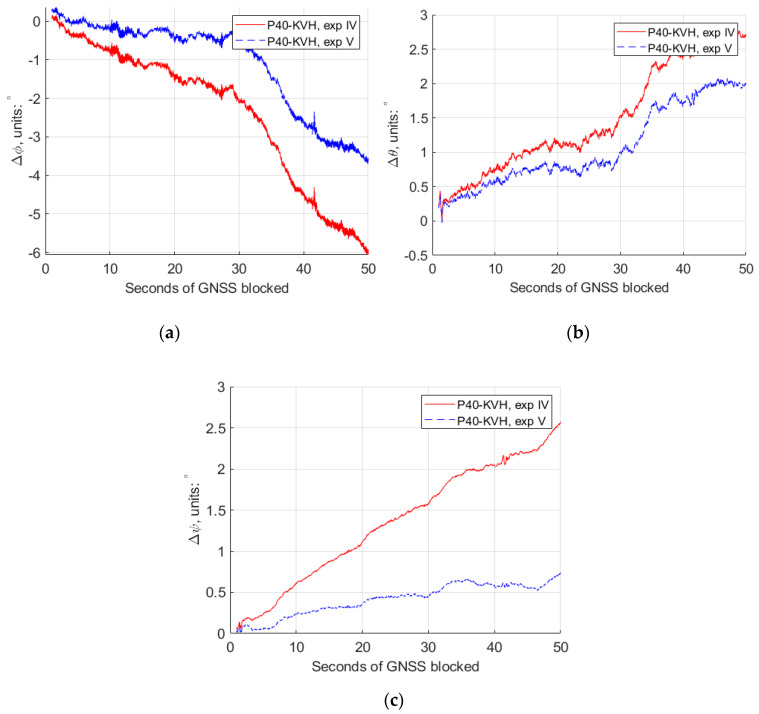
The roll, pitch and yaw attitude differences between GNSS/IMU-P40 (our program in a 15-state Kalman filter) and GNSS/KVH coupled navigation (IE software), with the conventional Kalman filter (experiment IV) and the modified Kalman filter (experiment V). (**a**) Roll differences; (**b**) pitch differences; (**c**) yaw differences.

For experiments IV and V, the 50 s GNSS missing part is in a straight line, and the conventional Kalman filter and the modified Kalman filter were applied, respectively, due to the different rate accelerometers retrieved from the “Huawei P40”. The comparison of the estimated trajectories at the experiment IV, V and the reference IMU is also shown in Figure 8. From these trajectories, we can see that the trajectory (blue) of the modified Kalman filter has a much weaker deviation trend compared to the conventional one (red). More detailed comparisons of the horizontal and vertical position differences, roll, pith and yaw differences are plotted as shown in Figure 9 and Figure 10.

Table 3 gives the numerical value from Figure 9 and Figure 10, at different second epochs of GNSS blocked at experiments IV and V. From Figure 9 and Figure 10, as well as Table 3, we can see that, with the modified Kalman filter (experiment V), the horizontal position accuracy was improved by 45%, from 379.0 m to 206.8 m, compared with the conventional ones (experiment IV) at the 50 s epoch of the GNSS observables missing. The attitude accuracy improvements by 40%, 26% and 71% of the roll, pitch and yaw, respectively, can also be obtained at that epoch.

**Figure 11:**
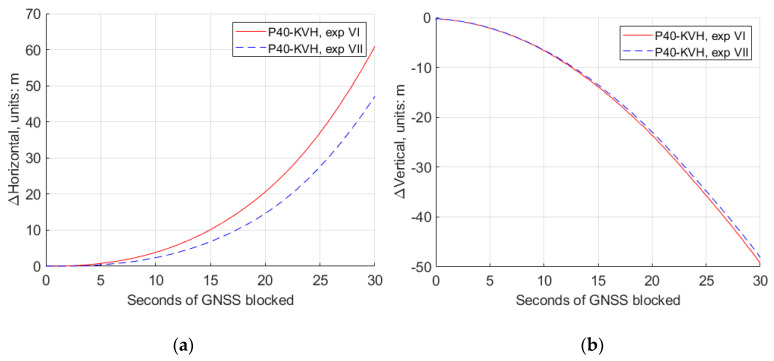
The horizontal and vertical position differences between GNSS/IMU-P40 (our program in a 15-state Kalman filter) and GNSS/KVH coupled navigation (IE software), with the conventional Kalman filter (experiment VI) and the modified Kalman filter (experiment VII). (**a**) Horizontal position differences; (**b**) vertical position differences.

**Figure 12:**
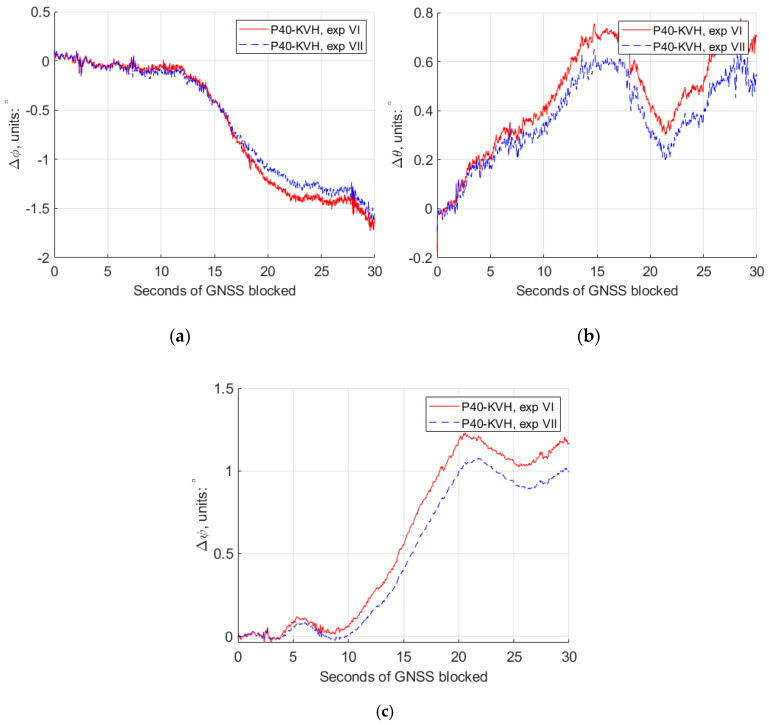
The roll, pitch and yaw attitude differences between GNSS/IMU-P40 (our program in a 15-state Kalman filter) and GNSS/KVH coupled navigation (IE software), with the conventional Kalman filter (experiment VI) and the modified Kalman filter (experiment VII). (**a**) Roll differences; (**b**) pitch differences; (**c**) yaw differences.

Similarly, for experiments VI and VII, the 30 s GNSS simulated observables missing were simulated at a turning part of the trajectory, and the conventional Kalman filter and the modified Kalman filter were applied, respectively, for different rate accelerometers retrieved from the “Huawei P40”. The comparison of the estimated trajectories at experiments VI and VII and the reference IMU are also shown in Figure 8, and the detailed comparisons of the horizontal and vertical position differences, roll, pith and yaw differences are plotted as shown in Figure 11 and Figure 12.

Table 4 gives the numerical value from Figure 11 and Figure 12, at different second epochs of GNSS blocked in experiments VI and VII. From Figure 11 and Figure 12 and Table 4, we can see that, with the modified Kalman filter (experiment VII), the horizontal position accuracy was improved by 23%, from 60.89 m to 46.73 m, compared with the conventional ones (experiment VI) at the 30 s epoch of the GNSS observables missing. The attitude accuracy improvements by 23% and 14% of the pitch and yaw, respectively, can also be obtained at that epoch.

Hence, the modified Kalman filter shows a better stability performance compared with the conventional Kalman filter.

## 5. Discussion

From this work, we can find that, using the modified Kalman filter, the performance of the GNSS/IMU-smartphone coupled navigation can provide significant improvement since it involves more IMU observations. This means that the method could be applied in many potential navigation or positioning applications, such as ITS (intelligent transportation system), smart cities, unmanned vehicles, etc.

Another issue that was not accomplished in this work, is the GNSS precise positioning based on the internal GNSS chips of smartphones. Ideally, the coupled navigation purely based on the smartphone embedded GNSS and IMU chips, will provide a powerful alternative approach to the other high-cost professional ones. However, the quality improvement of the GNSS raw observables tracked by the smartphones is still a challenging topic [16,17,18], as a result of the weak capacity of the duty cycling handling, internal tiny antenna and anti-electronic-inference.

## Figures and Tables

**Table 1:** The specifications of the IMUs used in the test [13,14,15].

IMUs	Accelerometers			Gyros		
	Bias Stability mg	Noise Density μg/sqrt (Hz)	Range g	Bias Stability °/s	Noise Density °/sqrt (hr)	Range °/sec
KVH 1750	0.05	12	±10	1.38 × 10^−5^	0.012	±490
ICM-20690 (P40)	40	100	±4	1	0.24	±250
Bosch-bmi160 (Play)	150	180	±4	3	0.42	±250

**Table 2:** The root mean square (RMS) statistics of the differences of the GNSS/IMU coupled solutions between the two smartphones (our program in a 15-state Kalman filter) and the “KVH 1750” (IE software).

Phone-KVH	ΔN m	ΔE m	ΔD m	ΔVn m/s	ΔVe m/s	ΔVd m/s	Δφ °	Δθ °	Δψ °
exp I (Play)	0.0921	0.0894	0.1209	0.0526	0.0559	0.0518	0.2222	0.1767	2.8137
exp II (P40)	0.0895	0.0941	0.1113	0.0605	0.0529	0.0567	0.1770	0.1700	3.4536
exp III (P40)	0.0906	0.0945	0.1104	0.0586	0.0523	0.0508	0.1658	0.1388	2.6432

**Table 3:** The difference values of the horizontal and vertical position, roll, pitch and yaw between GNSS/IMU-P40 (our program in a 15-state Kalman filter) and GNSS/KVH coupled navigation (IE software), at different epochs of the seconds of GNSS blocked in experiments IV and V.

P40-KVH	ΔHorizontal/m		ΔVertical/m		Δφ/°		Δθ/°		Δψ/°	
	exp IV	exp V	exp IV	exp V	exp IV	exp V	exp IV	exp V	exp IV	exp V
5 s	0.737	0.799	2.550	2.533	0.368	0.007	0.458	0.342	0.237	0.054
10 s	4.765	3.389	7.297	7.319	0.767	0.172	0.745	0.579	0.601	0.240
20 s	28.44	17.64	24.38	24.34	1.370	0.381	1.153	0.812	1.106	0.370
30 s	84.26	47.99	51.19	51.08	2.089	0.595	1.529	1.009	1.573	0.453
40 s	189.6	102.8	89.41	88.16	4.536	2.628	2.400	1.726	2.050	0.587
50 s	379.0	206.8	140.0	136.3	6.047	3.610	2.706	1.986	2.562	0.733

**Table 4:** The difference values of the horizontal and vertical position, roll, pitch and yaw between GNSS/IMU-P40 (our program in a 15-state Kalman filter) and GNSS/KVH coupled navigation (IE software), at different epochs of the seconds of GNSS blocked in experiments VI and VII.

P40−KVH	ΔHorizontal/m		ΔVertical/m		Δφ/°		Δθ/°		Δψ/°	
	exp VI	exp VII	exp VI	exp VII	exp VI	exp VII	exp VI	exp VII	exp VI	exp VII
5 s	0.788	0.395	−2.171	−2.144	−0.062	−0.072	0.206	0.174	0.086	0.049
10 s	3.796	2.391	−6.668	−6.567	−0.043	−0.076	0.403	0.337	0.061	0.001
20 s	20.56	14.58	23.65	−22.99	−1.223	−1.113	0.405	0.297	1.177	0.999
30 s	60.89	46.73	−49.29	−48.05	−1.550	−1.649	0.701	0.538	1.164	0.994
